# Effects of exogenous nicotinamide adenine dinucleotide (NAD+) in the rat heart are mediated by P2 purine receptors

**DOI:** 10.1186/s12929-016-0267-y

**Published:** 2016-06-27

**Authors:** Vladislav S. Kuzmin, Ksenia B. Pustovit, Denis V. Abramochkin

**Affiliations:** Department of Human and Animal Physiology, Lomonosov Moscow State University, Leninskie gory 1, building 12, Moscow, 119991 Russia; Department of Physiology, Pirogov Russian National Research Medical University, Ostrovitjanova 1, Moscow, 117997 Russia

**Keywords:** Heart, Rat, Action potential, NAD+, Purine receptors, Pulmonary veins

## Abstract

**Background:**

Recently, NAD+ has been considered as an essential factor, participating in nerve control of physiological functions and intercellular communication. NAD+ also has been supposed as endogenous activator of P1 and P2 purinoreceptors. Effects of extracellular NAD+ remain poorly investigated in cardiac tissue. This study aims to investigate the effects of extracellular NAD+ in different types of supraventricular and ventricular working myocardium from rat and their potential mechanisms.

**Methods:**

The standard technique of sharp microelectrode action potential recording in cardiac multicellular preparations was used to study the effects of NAD+.

**Results:**

Extracellular NAD+ induced significant changes in bioelectrical activity of left auricle (LA), right auricle (RA), pulmonary veins (PV) and right ventricular wall (RV) myocardial preparations. 10–100 μM NAD+ produced two opposite effects in LA and RA – quickly developing and transient prolongation of action potentials (AP) and delayed sustained AP shortening, which follows the initial positive effect. In PV and RV only AP shortening was observed in response to NAD+ application. In PV preparations AP shortening induced by NAD+ may be considered as a potential proarrhythmic effect. Revealed cardiotropic effects of NAD+ are likely to be mediated by P2 purine receptors, since P1 blocker DPCPX failed to affect them and P2 antagonist suramin abolished NAD + −induced alterations of electrical activity. P2X receptors may be responsible for NAD + −induced short-lasting AP prolongation, while P2Y receptors mediate persistent AP shortening. The latter effect is partially removed by PLC inhibitor U73122 showing the potential involvement of phosphoinositide signaling pathway in mediation of NAD+ cardiotropic effects.

**Conclusions:**

Extracellular NAD+ is supposed to be a novel regulator of cardiac electrical activity. P2 receptors represent the main target of NAD+ at least in the rat heart.

## Background

Nicotinamide adenine dinucleotide (NAD+) is ubiquitous in all living cells. Intracellular NAD+ is well known for more than a century as a crucial element of cell metabolism [1]. However, current investigations have demonstrated that NAD+ also plays essential role as an extracellular factor, participating in nerve control of various physiological functions and intercellular communication [2].

Several mechanisms of endogenous NAD+ release have been recognized in neuronal and non-neuronal tissues [3]. Regulated efflux of NAD+ through Cx43 hemichannels was shown in several mammalian cell types [4, 5]. According to the recent reports, the concentration of NAD+ in mammalian serum is around 100 nM but it can rise significantly due to the NAD+ efflux from damaged cells in pathological conditions [6].

More importantly, NAD^+^ may be released from nerve terminals together with classical neurotransmitters and produce physiological effects. For example, activation of sympathetic nerve terminals can induce the release of vesicles containing both noradrenaline and NAD+ [7–9], which subsequently contributes to the regulation of vascular and non-vascular smooth muscles, leading to either constriction or dilatation of blood vessels [10]. NAD+ also relaxes the colon musculature and displays properties of enteric inhibitory neurotransmitter [11, 12]. Active release, degradation and uptake of NAD+ have been described in rat brain synaptosomes. Moreover, released or exogenous NAD+ stimulates the postsynaptic neurons [13] and modulates the release of other neurotransmitters [7, 13]. Thereby, NAD+ is considered as a novel neurotransmitter contributing to the neurotransmission and neuromodulation in peripheral and central nervous system [3, 14].

NAD+ belongs to the group of natural purine compounds and thereby extracellular NAD+ may be considered as a purine receptors agonist. However, mechanisms of extracellular NAD+ action are very controversial due to the possible indirect activation of purine receptors [15]. Extracellular NAD+ might be degraded by nucleotide pyrophosphatases to AMP with subsequent cleavage by ecto-5’-nucleotidases to adenosine. Both compounds are well-known intrinsic P1 receptor agonists [16–18]. On the other hand, direct activation of P2 receptors by NAD+ has been also demonstrated in smooth muscles and other tissues. Recently, extracellular NAD+ has been shown to be an agonist of both P2Y and P2X receptors [2, 19, 20]. In blood vessels from various species NAD+ produced either vasodilation or vasoconstriction mediated by P1 and P2X receptors [10].

While the role of NAD+ in regulation of smooth muscle contractility has been explored extensively, effects of this compound remain poorly investigated in cardiac tissue. To our knowledge, only two earlier studies were dedicated to the cardiac effects of NAD+. We have recently reported that exogenous NAD+ alters contractility as well as action potential (AP) waveform in pacemaker and working atrial myocardium from rat [21, 22]. Nevertheless, effects of extracellular NAD+ in various regions of non-pacemaking myocardium have never been investigated in detail. Also, molecular mechanisms underlying cardiotropic effects of extracellular NAD+ have not been investigated.

Therefore, the purpose of present study was to reveal and compare effects of exogenous/extracellular NAD+ in supraventricular and ventricular working myocardium, and also to identify receptors and intracellular signaling pathways participating in mediation of NAD+ effects in the rat heart. Moreover, the present study demonstrate for the first time the effects of NAD+ in pulmonary veins (PVs) myocardium, which is involved in genesis of supraventricular arrhythmias [23].

## Methods

### Animals

All animal experiments were carried out in accordance with the Guide for the Care and Use of Laboratory Animals published by the US National Institutes of Health (NIH Publication No. 85–23, revised 1996). The experimental protocol was approved by Local Bioethics Committee of Moscow State University. Male Wistar rats weighing 250–300 g were used in the study (*n* = 98, 8 weeks old). Rats were held in the animal house for 4 weeks under a 12 h:12 h light:dark photoperiod in standard T4 cages prior to the experiment and fed *ad libitum*.

### Isolation of cardiac multicellular preparations

Rats were anesthetized with intraperitoneal injection of 80 mg/kg ketamine and 10 mg/kg xylazine. Heparin (1000 U/kg) was added to the anesthetics solution to prevent blood coagulation in the coronary vessels of the excised heart. The chest was opened and the heart was rapidly excised and placed into a bath with cold (+4 °C) Tyrode solution that contained (in mM): NaCl 118.0, KCl 2.7, NaH_2_PO_4_ 2.2, MgCl_2_ 1.2, CaCl_2_ 1.2, NaHCO_3_ 25.0, glucose 11.0, bubbled with carbogen, pH 7.4 ± 0.1.

The heart was rapidly rinsed by Krebs-Henseleit solution. Fragments of left auricle (LA), right auricle (RA) and right ventricular wall (RV) were isolated and pinned endocardial side up to the bottom of experimental chamber (3 ml) supplied with Tyrode solution at 10 ml min^−1^ (37.5 °C). Since all the types of preparations lacked intrinsic pacemaker activity, they were paced throughout the experiment with a pair of silver Teflon-coated electrodes (pacing rate – 3 Hz, pulse duration – 2 ms, pulse amplitude – 2 times threshold).

### Isolation of multicellular pulmonary veins preparations

Rats were anesthetized as described in the previous section. The chest was opened; the heart with lung lobes was rapidly excised and rinsed with physiological solution. To allow outflow of the solution, the outer edges of the lung lobes were trimmed. The left atrium was incised at the atrioventricular border and cannulated. Blood from the left atria and pulmonary veins was flushed out by injection of physiological solution. Next, fascia and pulmonary arteries were removed and preparation of isolated supraventricular region containing LA, pulmonary veins (PVs) and lung lobes was pinned in a preparation bowl. Finally, tubular PVs preparations were isolated from one or two lung lobes. Isolated PVs were cut along the axis and pinned in the experimental chamber inner side up. During the following experiments preparations were perfused and paced as described above.

### Microelectrode APs recording

After 1 h of equilibration, transmembrane potentials (APs) were recorded from LA, RA, RV and PVs with glass microelectrodes (30–45 MΩ) filled with 3 M KCl connected to a high input impedance amplifier Model 1600 (A-MSystems, Sequim, WA, USA). The signal was digitized and stored for further analysis using specific software (L-card, Russia). Stable impalements were maintained during the entire period of drugs application (up to 10 min). Changes in the APs duration at 90 % of repolarization (APD90) were determined in whole recordings using Minianalysis 3.0 software (Synaptosoft, USA).

NAD+ (1–100 μM) was administrated for 5 min after the 5 min of control recording. P1 receptors antagonist, various P2 receptors antagonists or phospholipases inhibitors were applied for 5 min prior and during whole period of NAD+ application.

### Drugs

NAD+, P1-blocker DPCPX was purchased from Sigma (St. Louis, MO, USA). Suramin, NF340, Iso-PPADs, U73122, FIPI were purchased from Tocris (Bristol, UK). Evans Blue and Coomassie Brilliant Blue G-250 were purchased from Thermo Scientific (Waltham, MA, USA).

### Statistical analysis

All data in the text and figures except the original recordings are presented as means ± SEM for n experiments. Statistica 6 (Dell Statistica, Tulsa, OK, USA) was used for statistical analysis of the data. Significance of NAD+ effects on registered parameters relative to the respective basal value of these parameters was evaluated by Wilcoxon test. The effects of NAD+ in the absence and presence of various blockers were compared by Mann–Whitney test. *P <* 0.05 was adopted as the level of statistical significance.

## Results

### Effects of NAD+ on AP waveform in isolated myocardial preparations

**Fig. 1:**
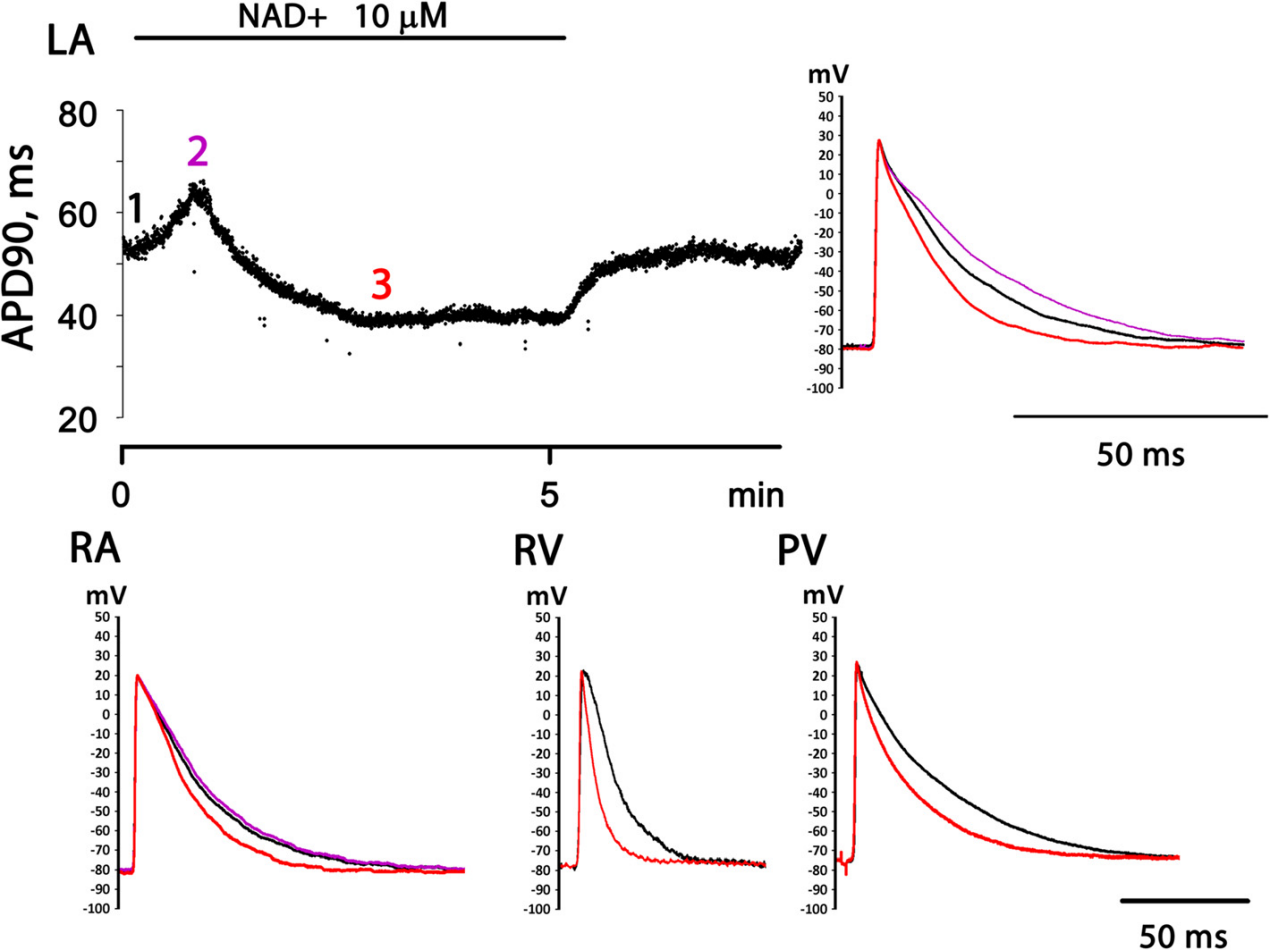
Electrophysiological effects of 10 μM NAD+ in various regions of the rat heart. Representative traces of APs recorded in left auricle (LA), right auricle (RA), right ventricular wall (RV) and pulmonary veins (PV) in control conditions and in the presence of NAD+ are superimposed. Black trace – control AP before NAD+ application. Red trace – AP at the moment of maximal negative effect (AP shortening) of NAD+. Magenta trace - AP at the moment of maximal positive effect (AP prolongation) of NAD+. Data from 4 separate representative experiments are shown. At the LA panel the time course of NAD+ effect on AP duration measured at 90 % repolarization level (APD90) is also shown (data from one representative experiment)

Exogenous NAD+ affected AP configuration in all tested types of atrial and ventricular myocardial preparations. In LA and RA application of NAD+ induced rapid transient increase in APD90, which was followed by sustained reduction of APD90 below the control values (Fig. 1). The latter effect was not fading until the start of washout. The extent of transient AP prolongation was very variable among experiments. Also, this effect of NAD+ was negligible in PVs and completely absent in RV (Fig. 1). Therefore, in PV and RV effect of NAD+ was delimited to AP shortening. Thus, NAD+ demonstrated complex dynamics of effect in atrial, but not in ventricular myocardium. Since AP prolongation was the most pronounced in LA, this type of preparations was selected for further investigation of this “positive” effect of NAD+.

**Fig. 2:**
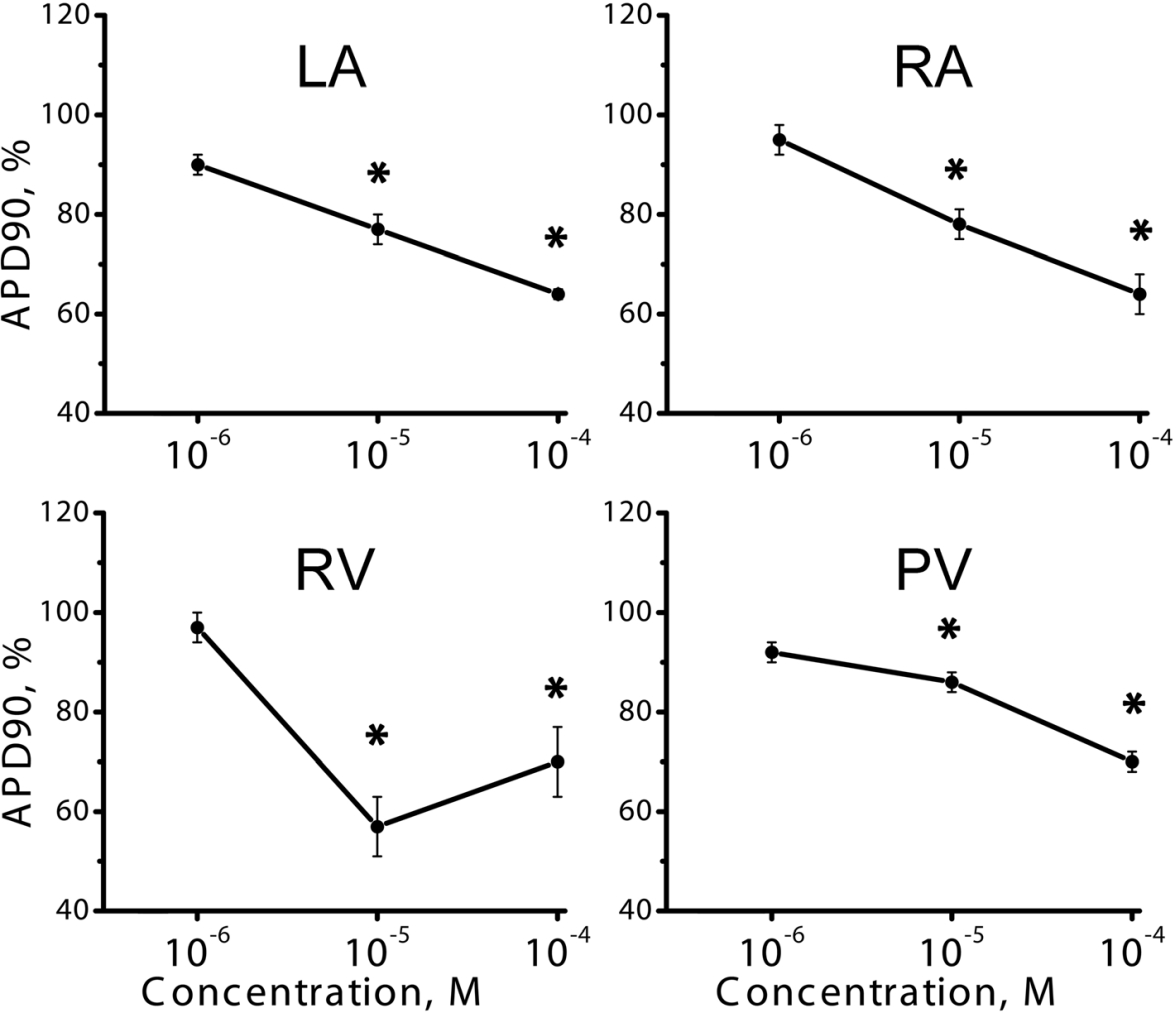
Dose dependency of NAD + −induced APD90 reduction in various isolated multicellular preparations of the rat heart. LA – left auricle, RA – right auricle, RV – right ventricle, PV – pulmonary veins. Relative APD90 is expressed in % of control APD90 measured before NAD+ application. * - significant difference of APD90 from the control value, *p* < 0.05, Wilcoxon test

Control APD90 in LA and RA preparations were 57.3 ± 5.2 ms (*n* = 15) and 50 ± 4.2 ms (*n* = 12), respectively. Application of 10 and 100 μM, but not 1 μM NAD+ induced significant transient APD90 prolongation in LA. Maximal increase of APD90 in response to 10 and 100 μM of NAD+ was by 17 % (66 ± 5 ms, *n* = 6, *p* < 0.05) and 19 % (68 ± 5 ms, *n* = 6, *p* < 0.05), respectively. Both in LA and RA, 10 and 100 μM NAD+ produced significant AP shortening after transient AP prolongation (Fig. 2). This “negative” effect of NAD+ was similarly expressed in RA and LA.

In ventricular preparations control APD90 was 33.8 ± 5.1 ms (*n* = 7). Application of 10 and 100 μM NAD+ led to substantial reduction of APD90 without any initial AP prolongation (Fig. 2). The effect was even greater than in the atrial preparations.

Paced PV preparations demonstrate atrial-like APs with fast upstroke front, overshoot and resting potential around −75 ± 3 mV (Fig. 1). Control APD90 in PVs was 72.5 ± 4 ms (*n* = 12). Similarly to normal atrial myocardium, PVs responded to administration of 10 and 100 μM NAD+ (*n* = 6) by significant (*p* < 0.05) reduction of APD90 (Fig. 2).

In all used types of preparations membrane potential was not affected by 10 and 100 μM NAD+.

### Effects of NAD+ in the presence of P1 and P2 receptors antagonists

**Fig. 3:**
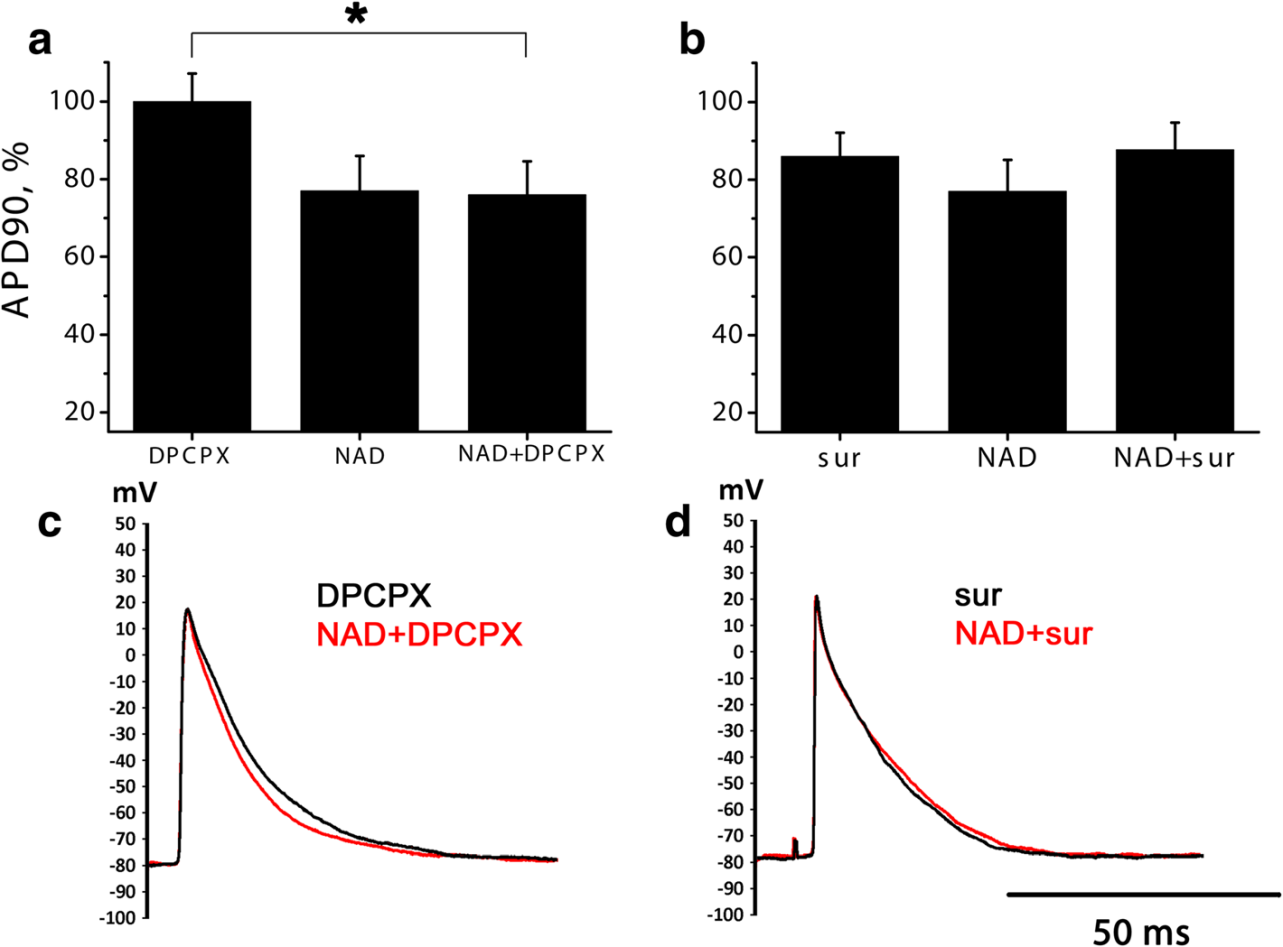
NAD + −induced AP shortening in the presence of selective antagonists of P1 and P2-purinoreceptors in LA preparations. **a**, **b** relative APD90 in the presence of P1 blocker DPCPX (0.1 μM, panel **a**) or P2 blocker suramine (sur, 10 μM, panel **b**), 10 μM NAD+ without blockers or NAD+ applied after DPCPX or suramine. APD90 is expressed in % of control APD90. NS – no significant difference, Mann–Whitney test. * - significant difference, Mann–Whitney test, *p* < 0.05. **c**, **d** representative traces of APs before and during NAD+ application in the presence of 0.1 μM DPCPX **c** or 10 μM suramine **d**

The ability of P1 and P2 selective antagonists to block electrophysiological effects of NAD+ were tested in LA preparations. P1 receptor antagonist DPCPX (0.1 μM) did not have any own influence on myocardial electrical activity, but also failed to suppress effects of NAD+ significantly (*n* = 10) (Fig. 3a, c). On the other hand, in presence of 10 μM P2-blocker suramin NAD+ did not produce significant changes of AP waveform (*n* = 6, *p* < 0.05, Fig. 3b, d). Thus, suramin abolished both transient positive and sustained negative effect of NAD+. It should be noted that suramin itself significantly shortened AP in LA preparations (Fig. 3b)

### Effects of NAD+ in the presence of P2Y11 receptors antagonist

**Fig. 4:**
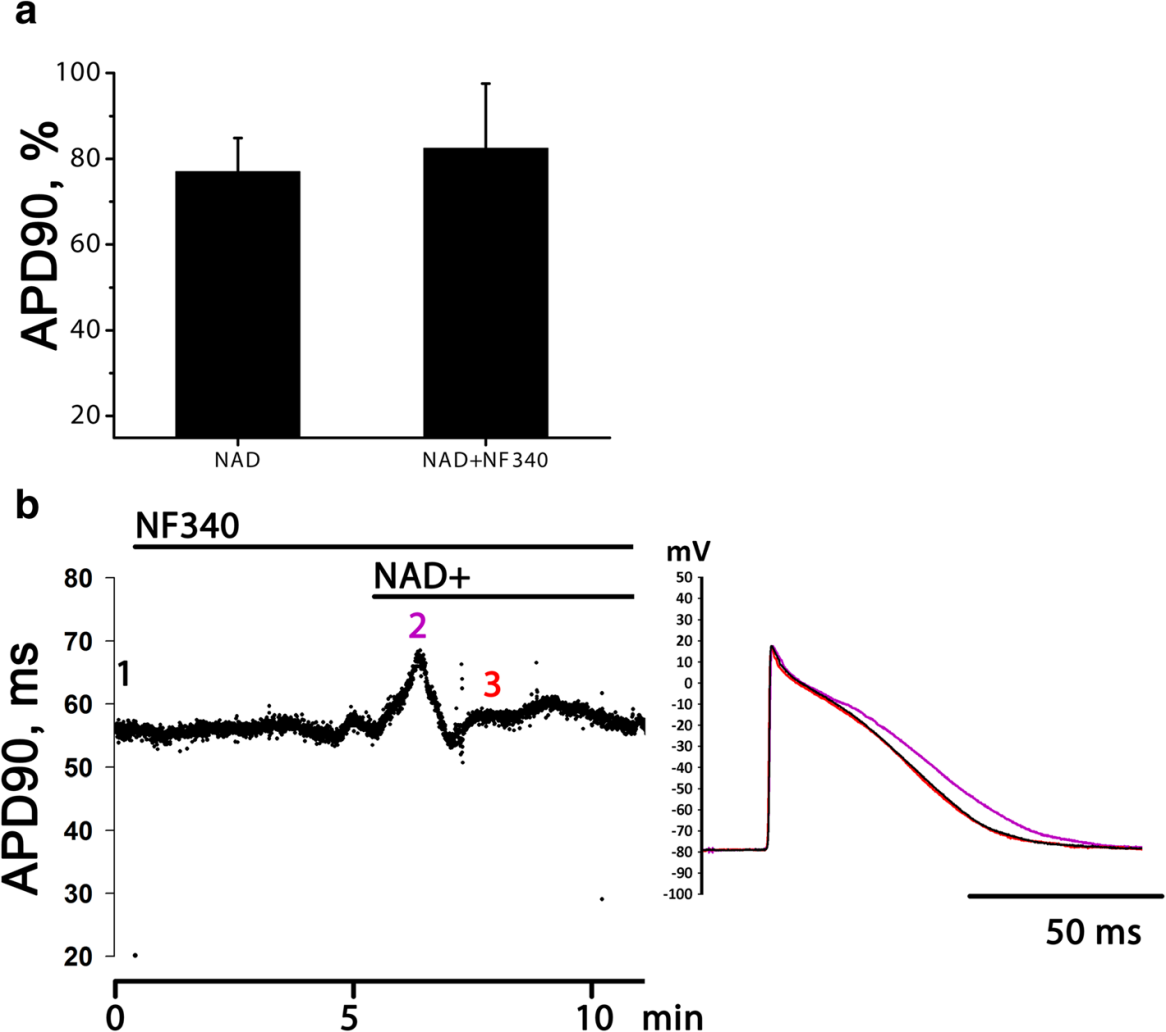
NAD + −induced AP shortening in the presence of selective P2Y11 receptor antagonist in LA preparations. **a** relative APD90 at the moment of maximal AP shortening induced by 10 μM NAD+ in normal conditions and in the presence of P2Y11 antagonist NF340 (10 μM). APD90 is expressed in % of control APD90. **b** time course of APD90 changes induced by NAD+ in the presence of NF340 and traces of APs recorded in the moments marked with numbers of respective color at the time course curve. This is the data from one of 2 experiments, where NF340 was effective

Effect of P2Y11 antagonist NF340 was also tested in LA preparations. Alteration of NAD + −induced (10 μM) APD90 decreasing by NF340 (10 μM) was very variable in our experiments. In 2 of 7 cases NF340 completely abolished NAD + −induced APD90 reduction with no effect on transient NAD + −induced AP prolongation (see example at Fig. 4b), while in 5 of 7 experiments NF340 failed to suppress negative effect of NAD+. Therefore, averaged effect of NF340 in LA was non-significant (Fig. 4a).

### Effects of NAD+ in the presence of P2X receptors antagonists

**Fig. 5:**
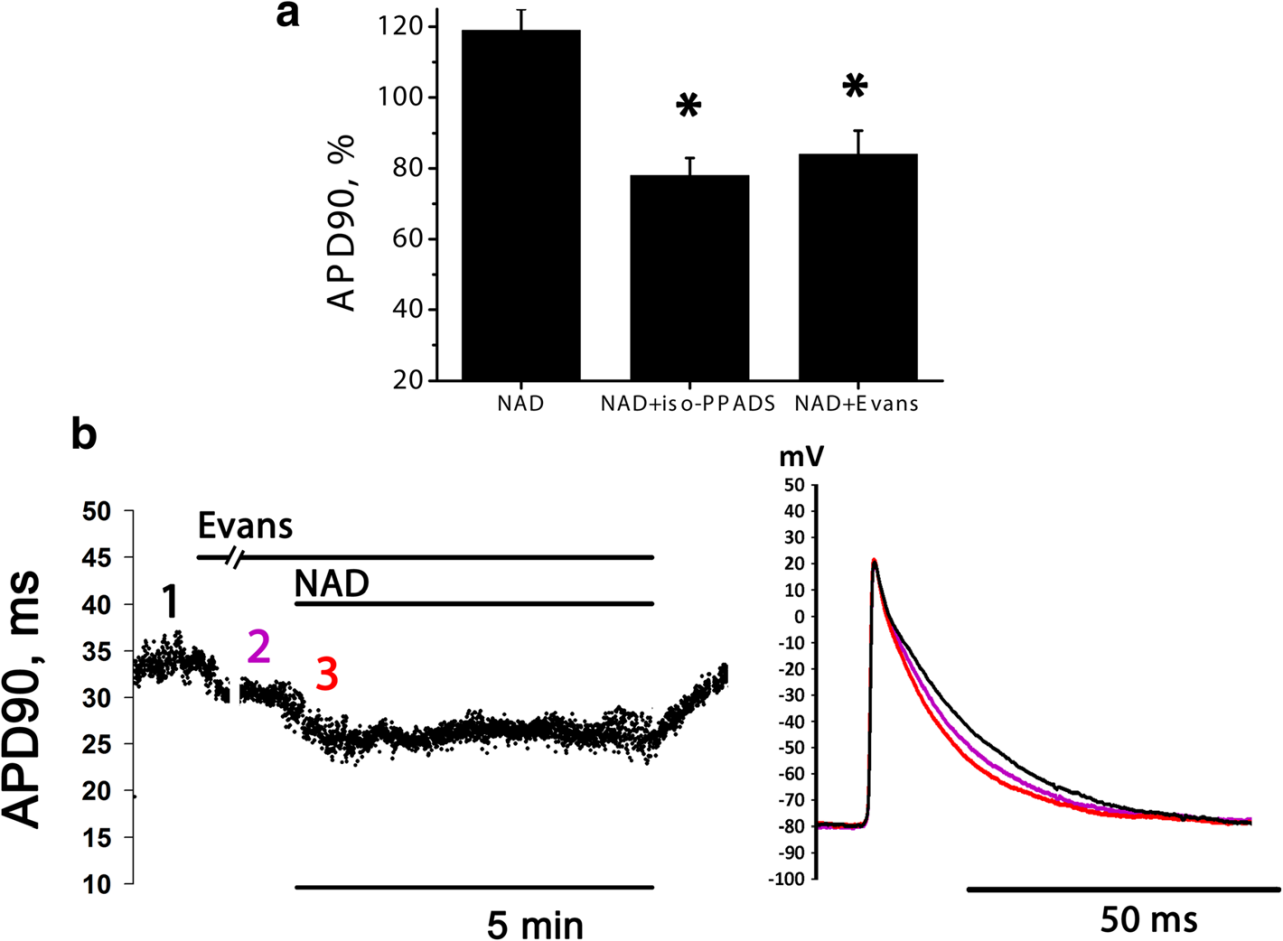
NAD + −induced transient AP prolongation in the presence of selective P2X antagonists in LA preparations. **a** relative APD90 measured at the moment of maximal AP prolongation in the absence of blockers and in the presence of P2X blockers iso-PPADS (1 μM) or Evans Blue (0.1 μM). APD90 is expressed in % of APD90 measured right before NAD+ application. * - significant difference from the effect of NAD+ alone, Mann–Whitney test, *p* < 0.05. **b** time course of APD90 changes induced by NAD+ in the presence of 0.1 μM Evans Blue and traces of APs recorded in the moments marked with numbers of respective color at the time course curve

Effect of P2X antagonists were also tested in LA preparations (Fig. 5). Two commercially available compounds were examined: iso-PPADs (1 μM, *n* = 7) and Evans Blue (0.1 μM, *n* = 5) – well known P2X receptor antagonists. Both compounds induced significant (*p* < 0.05) APD90 reduction in LA preparations by 10.2 % and 15 % respectively (data not shown).

Transient NAD + −induced (10 μM) AP prolongation was effectively reversed by both tested P2X-antagonists (see example for Evans Blue at Fig. 5b). Thus, positive effect of NAD+ on AP duration is likely to be attributed to P2X activation.

### NAD + −induced AP shortening in the presence of different phospholipases inhibitors

An attempt to identify the intracellular signal transduction elements mediating NAD + −induced APs shortening was made using the inhibitors of phospholipase C (U72122 0.1 μM) and D (FIPI, 0.1 μM) in LA preparations. Both inhibitors did not produce any changes in AP waveform prior to NAD+ application.

**Fig. 6:**
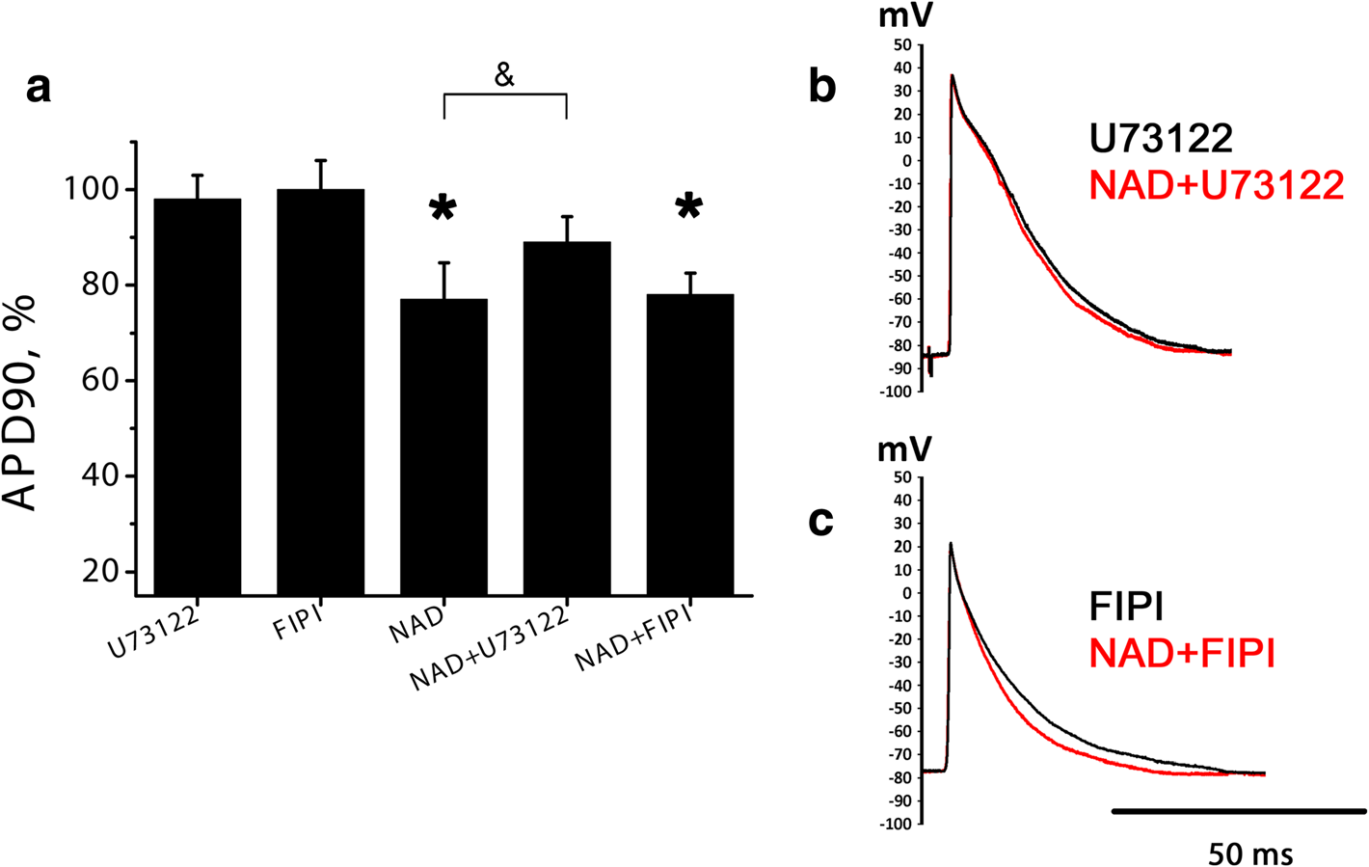
NAD + −induced AP shortening in the presence of phospholipases inhibitors. **a** relative APD90 at the moment of maximal AP shortening induced by 10 μM NAD+ in normal conditions and in the presence of PLC inhibitor U73122 (0.1 μM) or PLD inhibitor FIPI (0.1 μM). APD90 is expressed in % of control APD90. * - significant effect of NAD+, *p* < 0.05, Wilcoxon test. & - significant difference between the columns, *p* < 0.05, Mann–Whitney test. **b**, **c** representative traces of APs before and during NAD+ application in the presence of U73122 (**b**) or FIPI (**c**)

Negative effect of NAD+ was just partially, but significantly attenuated by U73122 (*n* = 8). NAD+ in combination with PLC inhibitor decreased APD90 only by 11 % (Fig. 6a, b). In additional pilot experiments U73122 in higher concentration (0.5 and 10 μM, both *n* = 3) was tested, but also caused just partial reduction of NAD+ effect (data not shown). Phospholipase D inhibitor FIPI (*n* = 5) failed to alter NAD + −induced AP shortening (Fig. 6a, c).

### Blockers of NO signaling pathway do not abolish NAD + −induced AP shortening

**Fig. 7:**
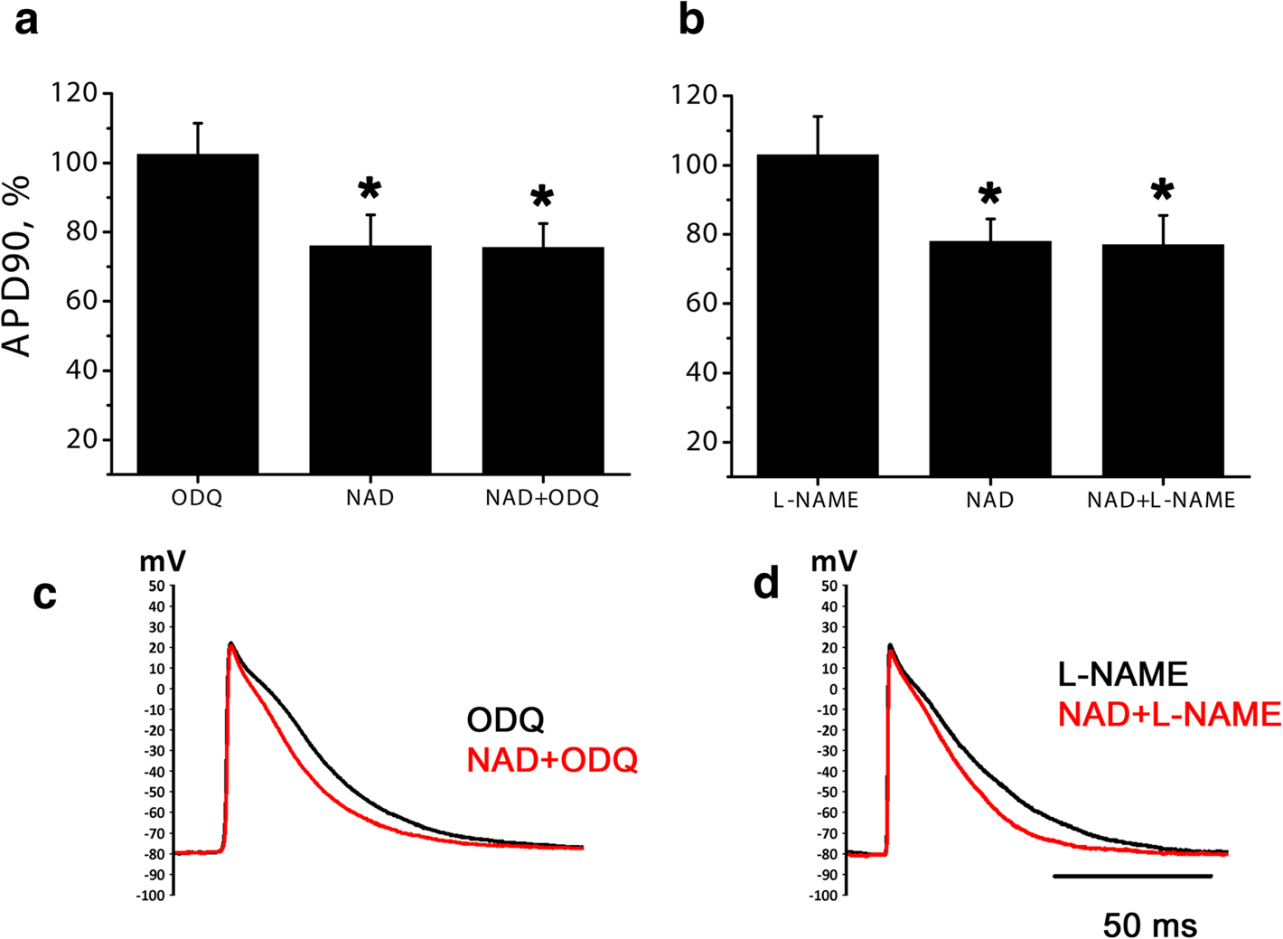
NAD + −induced AP shortening in the presence of NO signaling pathway blockers. **a** relative APD90 during the action of sGC inhibitor ODQ (5 μM) alone and at the moment of maximal AP shortening induced by 10 μM NAD+ in normal conditions and in the presence of ODQ. **b** relative APD90 during the action of NO-synthase inhibitor L-NAME (100 μM) alone and at the moment of maximal NAD+ effect in normal conditions and in the presence of L-NAME. APD90 is expressed in % of control APD90. * - significant difference from control, *p* < 0.05, Wilcoxon test. **c**, **d** representative traces of APs before and during NAD+ application in the presence of ODQ (**c**) or L-NAME (**d**)

Another putative mechanism of NAD+ negative effects involves increase of endogenous NO production via P2Y receptors, following activation of soluble guanylate cyclase (sGC) and increase in intracellular cGMP content. To check the possible role of NO signaling pathway in NAD + −induced AP shortening NAD+ was tested in the presence of sGC blocker ODQ (5 μM) and NO-synthase inhibitor L-NAME (100 μM). Being applied alone, both compounds tended to produce slight AP prolongation. However, in the presence of ODQ or L-NAME NAD+ was as effective as in normal conditions (Fig. 7). Therefore, blockers of NO signaling pathway failed to attenuate NAD + −induced AP shortening.

## Discussion

### Effects of NAD+ in the rat heart

The main objective of this study was to reveal electrophysiological effects of exogenous extracellular NAD+ in the various regions of the rat heart. To our knowledge, this is the first study demonstrating the alterations of AP waveform produced by NAD+ in RA, PVs and ventricular myocardium.

Exogenous extracellular NAD+ induced two opposite effects in LA and RA – quickly developing and transient prolongation of APs (“positive” effect) and delayed sustained AP shortening (“negative” effect), which follows the initial positive effect. In contrast to LA and RA, in both PVs and RV positive effect of NAD+ was absent and APD just decreased in response to NAD+ application. As mentioned above, the extent of positive effect varied among the experiments and was larger in LA in comparison with RA.

Negative effect was almost similar in all 3 types of supraventricular preparations, but more pronounced in ventricular myocardium. Both activation and suppression of I_CaL_ in response to ATP application have been demonstrated in cardiomyocytes from various mammalian species [24]. Similarly, suppression of I_CaL_ induced by NAD+ may partly explain APs shortening, although this assumption should be directly checked in further experiments with isolated rat myocytes. However, additional ionic mechanism including increase of repolarizing currents may underlie negative effect of NAD+.

It is known that pulmonary veins contain myocardial tissue that extends from the atria to the distal regions of the vessels. Starting from pioneering studies of Haissaguerre and coworkers [23], PVs myocardium was considered as the main source for AF initiation and maintenance. In the present investigation we have demonstrated the first evidence of AP shortening induced by NAD+ in PVs myocardium. It should be also mentioned that positive effect of NAD+ was negligible in PVs.

Since the normal level of NAD+ in mammalian serum appears to be by few orders lower than concentrations effective in cardiac muscle, we suppose that NAD+ is unlikely to regulate the cardiac function in normal conditions in hormone-like manner. However, several facts indicate that local concentrations of NAD+ may be much higher than 100 nM reported in swine serum. First, NAD+ is a co-mediator of NA and is released from sympathetic postganglionic nerve endings [7–9]. Second, necrosis of cardiomyocytes resulting from myocardial infarction leads to release of various intracellular compounds including NAD+ in the cardiac tissue thereby increasing the local NAD+ concentration within infarction zone. It has been well established, that supraventricular tachyarrhythmias, such as atrial fibrillation (AF) are associated with autonomic control abnormalities [25, 26]. For example, noradrenaline can initiate spontaneous activity in murine and canine PVs preparations [27, 28]. At the same time, NAD+ is released together with NA during sympathetic nerves stimulation [7–9]. NAD + −induced AP shortening and respective decrease of refractory period in atrial myocardium and PVs may be definitely considered as proarrhythmic effect, principally similar to the cholinergic shortening of refractory period [29]. In addition, extracellular NAD+ is known to elevate cytoplasmic Ca^2+^ concentration [20, 30–32] despite reduction of I_CaL_. Combination of spontaneous activity stimulation, AP shortening and intracellular Ca^2+^ mobilization is strongly proarrhythmic. Thus, extracellular NAD+ (including NAD+ excreted from sympathetic nerve endings) might act in concordance with NA and therefore enhance its proarrhythmic influence in PVs. Oppositely, in ventricular myocardium NAD+ is likely to be considered as potential antiarrhythmic factor, since AP shortening prevents development of early and delayed afterdepolarizations, responsible for onset of ventricular tachyarrhythmias. Thus, while physiological relevance of described cardiotropic effects of NAD+ is questionable, they seem to be important in pathophysiological conditions.

### Possible molecular mechanism of NAD+ cardiotropic effects

Extracellular NAD+ is considered nowadays as agonist of purine receptors and substrate for enzymes catalyzing the nucleotides breakdown [2, 3, 16]. Earlier studies supposed both P1- and P2-purine receptors as main targets for extracellular NAD+ in smooth muscle and other cell types. Also, simultaneous activation of multiple purine receptor subtypes by NAD+ has been suggested as a mechanism underlying the complex response in vascular and non-vascular smooth muscle tissues [10].

As described above, NAD+ induced complex positive–negative response in LA and RA, while in ventricular myocardium NAD+ produced only negative effect. We have tried to evaluate the role of purine receptor types in mediation of these effects. Both NAD + −induced transient AP prolongation and sustained shortening in LA was abolished by P2 blocker suramin, while P1-blocker DPCPX failed to antagonize these effects. It should be mentioned, that AP shortening induced by NAD+ shows remarkable similarity with the effects of ATP, well-known P2-agonist [24]. In rat cardiac myocytes the evidence of P1-mediated effects of ATP involving activation of acetylcholine-dependent inward rectifier (I_KACh_) has also been provided [33]. Therefore, stimulation of P1-receptors can produce effects similar to observed in our study. However, full ineffectiveness of DPCPX in our experiments allow to exclude this possibility. We suggest that P2 purine receptors are responsible for AP shortening induced by exogenous NAD+ in the rat heart.

The family of P2 receptors consists of ionotropic P2X receptors and metabotropic P2Y receptors coupled mainly to Gq/11-proteins. Activation of P2X receptors induces transient inward cationic current leading to increase in Ca^2+^ and Na^+^ intracellular level. The latter effect leads to alteration of Na/Ca-exchanger performance [34], which can also influence AP waveform. It is well known that all P2X receptors are characterized by quick desensitization [35]. Since transient increase of APs duration associated with NAD+ application was observed in our experiments, we checked the involvement of the P2X receptors in mediation of this effect. Expression of several P2X receptors types have been demonstrated in heart [36, 37]. On the contrary, sustained NAD + −induced AP shortening has been supposed to be P2Y-dependent.

In our experiments both iso-PPADS and Evans Blue [38, 39], widely used potent P2X blockers completely antagonized transient positive effect of NAD+ in rat LA preparations. These results allow to suggest the involvement of P2X receptors in the mediation of transient NAD+ positive effect in rat atrial myocardium. However, ionic and intracellular mechanisms underlying the P2X-dependent APs prolongation were beyond the scope of our study and deserve a special investigation.

NAD+ has been reported to be an endogenous P2Y11 agonist in several tissues, including colonic smooth muscle, immune and stem cells, [5, 40]. Cardiac tissue demonstrates the high level of P2Y11 receptors expression [36]. We have tried to clarify the role of P211Y receptors in cardiotropic effects of NAD+. P2Y11 antagonist abolished negative component of NAD+ effect only in 2 of 7 experiments. Therefore, particular subtype of P2Y receptors responsible for NAD+ effects in the rat heart remains questionable. Further investigations are needed, but complicated by lack of sufficiently selective P2Y subtype antagonists and potential variety of NAD+ targets in the heart.

Stimulation of P2Y-receptors leads to the activation of phosphoinositide signaling cascade involving activation of phospholipases. In earlier studies activation of G_q_/PI/PLC cascade has been suggested as the main mechanism underlying inhibitory effect of NAD+ in vascular and non-vascular smooth muscles. In central nervous system coupling of P2Y receptors to another phospholipase, PLD, has been shown [41]. In our experiments PLC inhibitor produced significant, but incomplete suppression of NAD + −induced AP shortening. In contrast to U73122, PLD inhibitor FIPI completely failed to attenuate NAD + −induced AP shortening. Therefore, our results favor the hypothesis considering involvement of PLC and phosphoinositide signaling cascade, but not PLD, in mediation of NAD+ negative effect.

However, involvement of other possible mechanisms of NAD+ effects needed to be checked. Coupling of P2Y receptors to the NO/cGMP cascade has been shown in several studies [42]. P2Y stimulation leads to increase in NO production, following stimulation of sGC and rise of intracellular cGMP content. cGMP stimulates phosphodiesterase 2, which is one of the prevalent phosphodiesterase isoforms in working myocardium of rabbit [43] and mouse [44], and therefore reduces intracellular cAMP content. Decrease of cAMP level leads to attenuation of I_CaL_ and subsequent AP shortening (see [45] for review). Therefore, we hypothesized that inhibitory effects of NAD+ in rat myocardium may be mediated by NO/cGMP pathway. However, neither L-NAME, which inhibits endogenous NO production, nor sGC blocker ODQ attenuated AP shortening produced by NAD+. Therefore, our results indicate that negative effect of NAD is mediated at least by G_q_/PI/PLC cascade, but not NO pathway.

### Limitations

The present study has several important limitations. First, some of the used inhibitors and blockers are not free of side-effects. Particularly, suramin, iso-PPADS and Evans Blue produced AP shortening, which can partially mask the negative effect of NAD+. Next, isolated multicellular preparations of myocardium have autonomic innervation, which could be affected by NAD+. Since there is no input from central nervous system driving the postganglionic parasympathetic neurons and sympathetic postganglionic fibers, we may assume the absence of evoked neurotransmitters release in the preparations. However, presence of non-quantal acetylcholine release, which does not require impulse activity of postganglionic neurons, has been shown recently in the rat heart [46, 47]. The possible regulation of this process by NAD+ and associated cholinergic effects cannot be excluded.

## Conclusions

The present study demonstrates significant changes of bioelectrical activity in LA, RA, PVs and ventricular myocardium from rat induced by exogenous extracellular NAD+. Revealed cardiotropic effects of NAD+ are likely to be mediated by P2 purine receptors. Particularly, P2X receptors may be responsible for NAD + −induced short-lasting AP prolongation, while P2Y receptors mediate subsequent persistent AP shortening. Intracellular pathway mediating NAD+/P2Y effects in the rat heart remains unclear, however we suppose the involvement of Gq/PI/PLC signaling cascade. Thus, NAD+ should be considered as a novel regulator of cardiac electrical activity and endogenously produced NAD+ may take part in autonomic control of a cardiac function.

## Abbreviations

AF, atrial fibrillation; AP, action potential; APD90, action potential at 90 % repolarization level; cAMP, cyclic adenosine monophosphate; cGMP, cyclic guanosine monophosphate; Cx43, connexin 43; LA, left auricle; L-NAME, NG-Nitro-L-arginine methyl ester hydrochloride; NA, noradrenaline; NAD+, Nicotinamide adenine dinucleotide; NO, nitric oxide; PLC, phospholipase C; PLD, phospholipase D; PVs, pulmonary veins; RA, right auricle; RV, right ventricular wall
